# The Systematic Medical Appraisal Referral and Treatment Mental Health Project: Quasi-Experimental Study to Evaluate a Technology-Enabled Mental Health Services Delivery Model Implemented in Rural India

**DOI:** 10.2196/15553

**Published:** 2020-02-27

**Authors:** Pallab K Maulik, Siddhardha Devarapalli, Sudha Kallakuri, Amritendu Bhattacharya, David Peiris, Anushka Patel

**Affiliations:** 1 George Insitute for Global Health New Delhi India; 2 University of New South Wales Sydney Australia; 3 George Institute for Global Health University of Oxford Oxford United Kingdom; 4 George Institute for Global Health Sydney Australia

**Keywords:** mental health services, mHealth, rural, India, mental disorders, primary health care

## Abstract

**Background:**

Although around 10% of Indians experience depression, anxiety, or alcohol use disorders, very few receive adequate mental health care, especially in rural communities. Stigma and limited availability of mental health services contribute to this treatment gap. The Systematic Medical Appraisal Referral and Treatment Mental Health project aimed to address this gap.

**Objective:**

This study aimed to evaluate the effectiveness of an intervention in increasing the use of mental health services and reducing depression and anxiety scores among individuals at high risk of common mental disorders.

**Methods:**

A before-after study was conducted from 2014 to 2019 in 12 villages in Andhra Pradesh, India. The intervention comprised a community antistigma campaign, with the training of lay village health workers and primary care doctors to identify and manage individuals with stress, depression, and suicide risk using an electronic clinical decision support system.

**Results:**

In total, 900 of 22,046 (4.08%) adults screened by health workers had increased stress, depression, or suicide risk and were referred to a primary care doctor. At follow-up, 731 out of 900 (81.2%) reported visiting the doctor for their mental health symptoms, compared with 3.3% (30/900) at baseline (odds ratio 133.3, 95% CI 89.0 to 199.7; *P*<.001). Mean depression and anxiety scores were significantly lower postintervention compared with baseline from 13.4 to 3.1 (*P*<.001) and from 12.9 to 1.9 (*P*<.001), respectively.

**Conclusions:**

The intervention was associated with a marked increase in service uptake and clinically important reductions in depression and anxiety symptom scores. This will be further evaluated in a large-scale cluster randomized controlled trial.

## Introduction

### Background

The Global Burden of Disease study estimates that about 7.1% of total disability-adjusted life years lost are because of mental and substance use disorders [1]. Recent surveys from India estimate that around 10% of the population (150 million) experience depression, anxiety, alcohol, and substance use disorders requiring mental health care [2]; however, only 15% to 25% receive any treatment in low- and middle-income countries (LMICs), such as India [3]. Likely contributors to this gap are poor mental health awareness, stigma associated with mental disorders, few trained mental health professionals, and limited relevant health care services [4,5]. Rural areas specifically lack mental health services, and awareness is low. As major increases in mental health workforce capacity are infeasible, alternate strategies using existing health care providers are needed. One such strategy involves empowering existing workforce cadres through the provision of training and electronic decision support systems (EDSSs) to facilitate evidence-based mental health care [6-10]. Although data from LMICs are limited, some interventions involving digital health and those involving task sharing between doctors and nonphysician health workers have shown promise [11,12]. Strategies to increase mental health awareness and reduce stigma have also been shown to be critical to complement clinical approaches [13,14].

The Systematic Medical Appraisal Referral and Treatment (SMART) Mental Health project was conducted in the West Godavari district of rural Andhra Pradesh, India. The intervention used the principles of task sharing supported by a technology-enabled mental health services delivery model for screening, diagnosing, and managing common mental disorder*s (CMDs)*—defined here as stress, depression, and increased suicide risk.

### Objective

The key objective was to evaluate the acceptability, feasibility, and preliminary effectiveness of the intervention in increasing the use of mental health services and reducing depression and anxiety scores using a pre-post study design [14]. The effectiveness data are reported here. Findings from a mixed methods process evaluation will be reported separately.

## Methods

### Project Site and Inclusion Criteria

The project was implemented in 12 villages served by 3 primary health care centers (PHCs) selected purposively based on a maximum radial distance of 35 km from the field office and an available doctor. All eligible villages were listed, with 4 villages from each PHC selected at random. The village eligibility criterion was the availability of Accredited Social Health Activists (ASHAs) proportionate to the population as designated by the government (ie, 1 ASHA per 1000 population). ASHAs are lay female village health workers who receive basic health care training with a primary focus on maternal and child health. Community members targeted for the intervention were all individuals aged 18 years or older, who could provide consent and who did not have any physical illness that led to mobility restrictions and prevented access to PHCs.

### Duration

An initial formative phase [15] was conducted in which screening and treatment algorithms developed for the EDSS were tested iteratively using simulated data, and mock clinical data were validated against a psychiatrist’s diagnosis. Following this, the intervention was implemented between November 2015 and November 2016, with postintervention data collection being implemented between December 2016 and February 2017.

### Prestigma Campaign Data Collection

Trained interviewers collected specific data on stigma perceptions of the community in 2 villages, which were selected purposively based on distance from the field office and population size [16]. Owing to limited resources, the evaluation of the antistigma campaign was limited to just 2 villages.

### Baseline Data Collection

Trained interviewers conducted a baseline survey in all villages using a purpose-built data collection application on a mobile tablet device, with results reported separately [17]. Questions focused on sociodemographic status; major life events, such as loss of employment and death in the family; social support networks; past history of CMDs and its treatment; family history of mental disorders; and perceptions about stigma related to mental health. Those who scored 10 or greater on either the 9-item Patient Health Questionnaire (PHQ-9) [18] or 7-item Generalized Anxiety Disorder (GAD-7) [19] or scored 1 or greater on the self-harm–related question of the PHQ-9 were considered to be *screen positive* (hence at an increased risk of CMD) and were advised to seek care from the primary care doctor or a mental health specialist. Anyone identified with severe depression (a score of ≥15 on either the PHQ-9 and/or GAD-7 [20]) or increased suicide risk (a score ≥1 on the self-harm–related question of PHQ-9) was specifically referred for immediate care, and family members were notified after obtaining consent from the interviewee.

### Intervention

**Figure 1:**
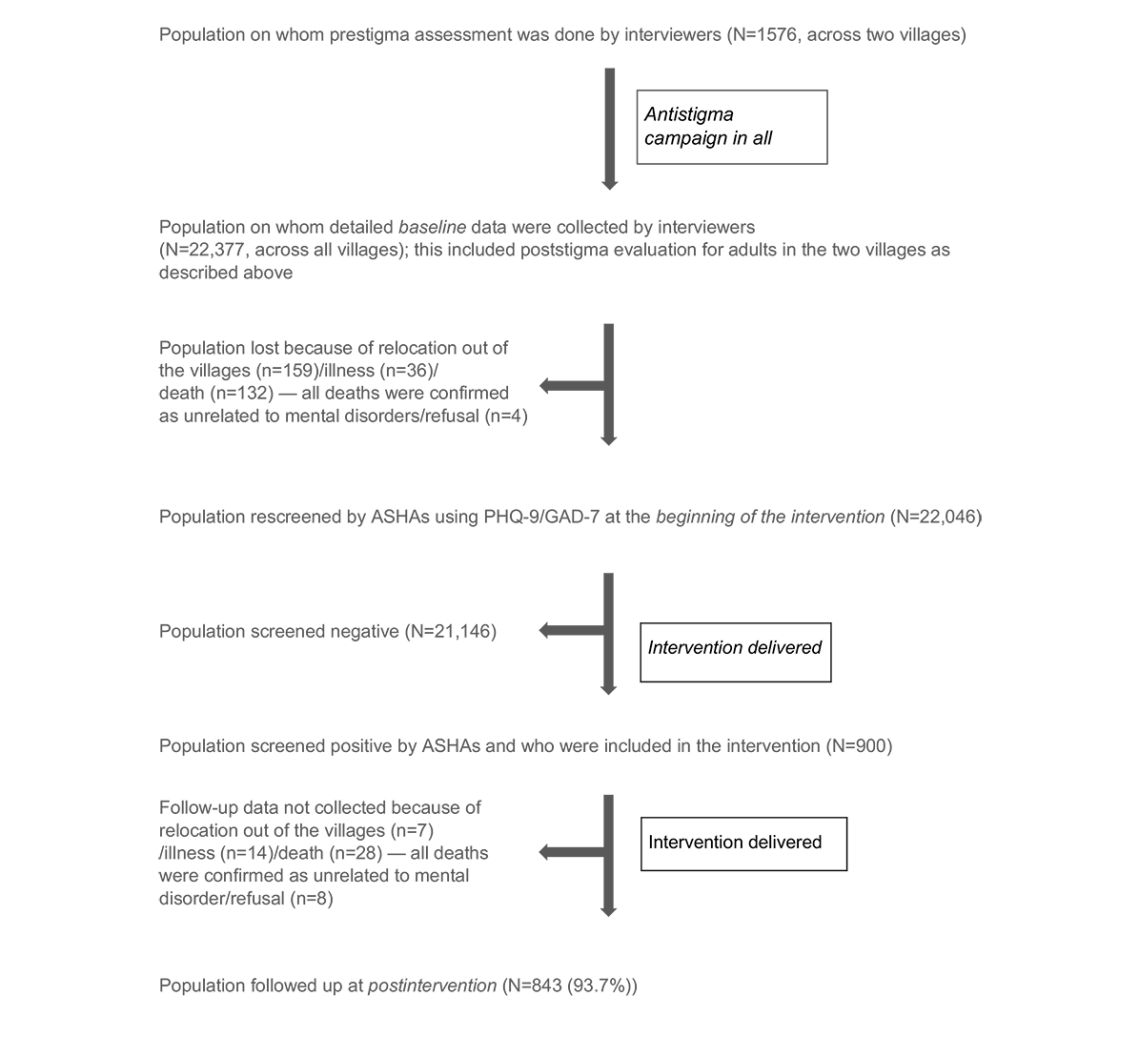
Diagram showing population contacted and interviewed at each stage. ASHA: Accredited Social Health Activist; GAD-7: 7-item Generalized Anxiety Disorder; PHQ-7: 7-item: Patient Health Questionnaire.

The intervention was developed and tested during formative work [15] using mixed methods. In brief, the intervention comprised (1) an antistigma campaign, (2) training of ASHAs to screen for CMDs using the PHQ-9 and GAD-7 on Android tablets and to refer high-risk individuals to the PHC, (3) training of doctors to implement management guidelines using point-of-care decision support also using Android tablets, and (4) a recall system for ASHAs and doctors to follow-up patients. A cloud-based electronic medical record system (OpenMRS) was used to store clinical information and allow data to be shared between the ASHAs and doctors (Figure 1).

#### The Antistigma Campaign

This comprised multimedia approaches, involving printed materials, videos, drama, and a house-to-house campaign, and has been described separately in detail [16,21]. It was initially rolled out across all villages following the prestigma data collection and before the baseline survey. The campaign was assessed using mixed methods in the 2 villages, where prestigma data were collected. The mental health services delivery component was implemented subsequent to the antistigma campaign following the baseline survey after training the primary health workers.

#### Accredited Social Health Activist Training in Screening for Common Mental Disorders

Research staff provided training to 40 ASHAs and 5 medical officers from the 3 PHCs. The training focused on identification and management of CMDs. The ASHAs were trained for 2 weeks using videos, presentations, and discussions of case vignettes. ASHAs were asked to screen the entire adult population in their villages using Telugu versions of PHQ-9 and GAD-7 to identify screen-positive individuals, without access to the data independently collected during the baseline survey. Both the PHQ-9 and GAD-7 identified people at mild, moderate, and severe risk of depression and anxiety based on scores 5 to 9, 10 to 14, and 15 or greater, respectively [18-20]. The EDSSs required those who scored between 5 and 9 on either the PHQ-9 or GAD-7 to be reinterviewed 2 weeks later to determine if they had become screen positive. Individuals referred to the doctors based on their *screen positive* status were seen either at the PHCs or at health camps organized in the villages.

#### Doctor Training

The PHC doctors were trained in the use of the World Health Organization Mental Health Gap Intervention Guide (mhGAP-IG) by a trained psychiatrist, using presentations and case vignettes [22]. Three modules from the mhGAP-IG tool were used—*depression, suicidal intent or self-harm, and other emotional or medically unexplained complaints.* Decision support algorithms were developed based on the stress, depression, and suicidal modules of the mhGAP-IG guidelines and deployed on 7-inch Android tablets for the doctors to use [22]. One person could have comorbid diagnoses. Those with emotional stress/mild depression were counseled, and those with moderate depression/suicide risk were counseled and/or prescribed antidepressants. Clinical symptoms suggestive of psychotic features, mania/hypomania, bereavement, and alcohol or substance use were checked as indicated in the mhGAP-IG module on depression. Counseling included discussions on ways to overcome stressors and involve one’s social support systems and were based on mhGAP-IG guidelines. Individuals diagnosed with moderate depression who could not afford to purchase antidepressants were also referred to the district hospital for receipt of free drugs. Individuals with bipolar disorder or alcohol or drug use or psychotic symptoms (as assessed by their symptom profiles) were immediately referred to the district hospital for specialist mental health care. Doctors were provided support by the field staff in navigating the EDSS in the initial stages, but this reduced over time. Any doubts that doctors had about specific questions related to the mhGAP-IG were also clarified by the research team.

#### Follow-Up of Patients

ASHAs followed up screen-positive individuals based on a prioritization list programmed in their tablet devices. They asked specific questions that were predetermined based on the patient’s status, as shown in the prioritization list. The questions checked about follow-up with doctor (or reasons for not doing so), treatment adherence as per doctor’s advice, follow-up with specialist if advised by the doctor, mental well-being, stressors, and social support systems. Interactive voice response messages facilitated the process by sending tailored prerecorded messages to screen-positive individuals reinforcing advice provided by ASHAs or doctors, to ASHAs ensuring follow-up of individuals, and to doctors reminding them to schedule health camp visits. These were sent as voice messages during the whole intervention period.

### Postintervention Data Collection

Individuals who were screened positive by ASHAs were followed up postintervention using questionnaires administered by trained interviewers to collect outcome data.

### Outcomes

The primary outcome was the proportion of individuals identified by ASHAs at increased risk of CMDs, who accessed mental health services from their PHC doctors at least once over the intervention period (between November 2015 and November 2016), compared with the proportion who reported accessing mental health services from any health provider at any time before the intervention. Secondary outcomes included change in depression and anxiety scores using validated questionnaires (PHQ-9 and GAD-7) [18,19] and changes in proportions of those with moderate or severe depression/anxiety (reported in this paper) and scores on knowledge, attitude, and behavior related to mental health and stigma perception related to help-seeking reported in a previous paper [16,21].

### Data Management and Statistical Analyses

All data were captured electronically and stored on secure servers at the George Institute office, Hyderabad. All tablets and servers were password protected. Data on tablets could be accessed by a user-defined log-in, and only the administrator had the ability to conduct data quality checks and rectify any errors. Deidentified data extracts were generated for statistical analyses.

### Sample Size

We anticipated that 12 villages would have a population of around 27,000 adults aged 18 years or older eligible to receive the intervention. On the basis of previous work where we obtained a response rate of 84% [23], we conservatively assumed 75% (approximately 19,500 participants) would participate. It was estimated that 15% of consenting participants at baseline would be at increased risk of CMD. This equates to approximately 3000 to 4000 individuals. On the basis of past research, we assumed that 10% of screen-positive individuals would have sought medical care for their symptoms in the previous 12 months at baseline [3]. A previous study that focused on the provision of mental health services in India using primary care workers had found an intraclass correlation coefficient (ICC) of 0.03 using mental health service providers as the unit of clustering [24]. Unlike the study [24] that used PHCs as clusters to assess the behavioral intervention, this study evaluated behavioral intervention using ASHAs as clusters. Hence, we assumed a more conservative ICC of 0.1, as we expected greater correlation among individuals cared by a particular ASHA. For statistical purposes, ASHAs were considered as the clusters for analyses because ASHAs were the main primary health workers who screened the community, ensured follow-up with doctors, and routinely followed patients for treatment adherence. On the basis of these assumptions, the study had 80% power at an alpha of .05 to detect a relative increase of mental health care utilization (primary outcome) by at least 20%, at follow-up, with 38 clusters and 80 individuals in each cluster. 

### Analysis

An *a priori* statistical analysis plan was developed (Multimedia Appendix 1). The primary outcome was analyzed at the patient level after adjusting for clustering using generalized mixed effects modeling, where ASHAs were the random effects and the pre- and postintervention assessments were the fixed effects. Initial models checked the effect of sociodemographic variables on mental health services use, based on prior research [25], along with the pre-and postintervention status. Age was categorized into less than 30 years, 30 to 59 years, and 60 years or older; gender, as male/female; marital status, as currently married, never married, and separated/divorced/widowed; education, as educated/not educated; occupation, as working/not working (Multimedia Appendix 2). Only the significant covariates (*P*<.05) were included in the final multivariate model to obtain adjusted estimates for mental health services use. Nonlinear Newton Raphson optimization was used in the model to aid convergence in the generalized mixed linear model.

Sensitivity analyses were performed for the primary outcome based on responses obtained from those individuals who were screened positive by interviewers at baseline but were subsequently not found to be screen positive when ASHAs rescreened them and are reported in Multimedia Appendix 2. For the secondary outcome, both proportions with moderate/severe depression/anxiety (scored ≥10 on either the PHQ-9 or GAD-7) and mean depression and anxiety scores among those who had scored 10 or greater on either the PHQ-9 or GAD-7 at the beginning of the intervention were also compared with the proportions and scores at postintervention after adjusting for clustering by ASHAs using mixed models as mentioned earlier.

### Ethics and Other Approvals

The Independent Ethics Review Committee of the Centre for Chronic Disease Control, New Delhi, approved the study. Participants provided written informed consent. Approvals were also obtained from the Health Department, Government of Andhra Pradesh, and each local village administration. The authors assert that all procedures contributing to this work comply with the ethical standards of the relevant national and institutional committees on human experimentation and with the Helsinki Declaration of 1975, as revised in 2008.

### Role of Funding Source

The funders had no role in the study design, data collection, interpretation of results, and reporting.

## Results

### Baseline Screening and Sociodemographic Characteristics

The baseline survey conducted by the trained interviewers included 22,377 of 27,867 adults (80.3% of the total estimated eligible population). The ASHAs screened 22,046 adults, who were available for interview and consented. They identified 900 (4.1%) adults as screen positive based on the study criteria (Figure 1). Of 900 adults, 150 had also been identified as screen positive by the interviewers at baseline. The concordance between ASHA and interviewer screening was low (kappa=0.11; 95% CI 0.08 to 0.13).

At postintervention, 843 of the 900 adults identified as screen positive by ASHAs were reassessed by independent interviewers. In total, 28 of the 57 adults lost at follow-up had died, and all were because of causes unrelated to mental disorders (Figure 1).

Table 1 compares the sociodemographic characteristics of the population screened by ASHAs and those who were screened positive. Compared with the screen-negative population, those screened positive were older and more likely to be women, separated/divorced/widowed, and with no formal education, and all of these differences were statistically significant (*P*<.001).

**Table 1:** Sociodemographic and health characteristics of the study population who were screened by Accredited Social Health Activists (N=22,046).

Characteristic		Screened negative (n=21,146)		Screened positive and received a formal diagnosis by the doctor (n=242)		Screened positive but did not receive a formal diagnosis by the doctor (n=489)		Screened positive but did not visit the doctor (n=169)	
**Age (years)**									
	Mean (SD)		41.8 (15.83)		47.8 (15.79)		53.3 (15.30)		49.4 (16.26)
	Range		18-98		19-90		18-90		19-92
**Gender, n (%)**									
	Female		11,395 (53.89)		167 (69.0)		347 (71.0)		113 (66.9)
	Male		9751 (46.11)		75 (31.0)		142 (29.0)		56 (33.1)
**Occupation, n (%)**									
	Housewife/retired		724 (3.42)		2 (0.8)		5 (1.0)		0 (0.0)
	Organized sector^a^		4998 (23.64)		61 (25.2)		89 (18.2)		44 (26.0)
	Unorganized sector^b^		11,642 (55.06)		131 (54.1)		230 (47.0)		79 (46.7)
	Others^c^		3782 (17.89)		48 (19.8)		165 (33.7)		46 (27.2)
**Education, n (%)**									
	Graduate/postgraduate		1055 (4.99)		2 (0.8)		4 (0.8)		3 (1.8)
	High school		4288 (20.28)		22 (9.1)		28 (5.7)		11 (6.5)
	Primary school		8922 (42.19)		88 (36.4)		184 (37.6)		73 (43.2)
	No school		6706 (31.71)		130 (53.7)		273 (55.8)		81 (47.9)
	Others^d^		175 (0.83)		0 (0.0)		0 (0.0)		1 (0.6)
**Marital status, n (%)**									
	Currently married		16,982 (80.31)		197 (81.4)		354 (72.4)		125 (74.0)
	Never married		2085 (9.86)		7 (2.9)		10 (2.0)		2 (1.2)
	Separated/divorced/widowed		2079 (9.83)		38 (15.7)		125 (25.6)		42 (24.9)

^a^All regular salaried jobs were part of the organized sector.

^b^Agricultural laborer, manual laborer, skilled worker, farmer, and business are reported under the unorganized sector.

^c^Includes students, those searching for jobs, and those unable to work because of illness and old age.

^d^Those pursuing vocational training.

### Mental Health Services Use

Among those screened positive (n=900) and followed up at the end of the study (n=843), self-reported prior use of mental health services at any time in the past was 3.3% (30/900) at baseline. At the end of the intervention phase, this increased to 81.2% (731/900, odds ratio [OR] 133.3, 95% CI 89.0 to 199.7; *P*<.001). Among the different covariates predicting mental health services use, only marital status was found to be significant at *P*<.05 (Multimedia Appendix 2)*.* Marital status was included in the final multivariate model along with the intervention. The OR for mental health service use adjusted for the intervention and marital status was 137.8 (95% CI 91.4 to 207.7; *P*<.001).

In total, 731 of 900 (81.2%) screen-positive individuals accessed mental health care from the PHC doctors, with 716 individuals visiting the doctor at the health camps and only 15 seeking care at the PHC. Of the 731 individuals who sought care, 514 (70%) were female and 242 (33.1%) were clinically diagnosed with a mental illness by the doctor as per the mhGAP-IG tool. Compared with those who did not receive a clinical diagnosis from the doctor, individuals who received a clinical diagnosis were younger or married (Table 1). Of those assessed, almost 50% (152/303) were suffering from emotional stress, mild/moderate depression, or suicide risk (Table 2).

Of 242 individuals who had a clinical condition following the doctor’s assessment (Table 2), 94 (38.8%) attended a second doctor visit, and 116 (47.9%) of them had residual symptoms requiring further treatment. Of 242 individuals, 10 (4.1%) attended a third doctor visit, with 3 requiring further treatment. The ASHAs were able to follow up with 888 of the 900 (98.7%) screen-positive individuals at least once during their routine home visits and reinforce treatment adherence.

**Table 2:** Outcome of clinical assessment of patients by primary care doctors.

Clinical conditions	Total clinical conditions (N=303)^a^, n (%)
Emotional stress	91 (30.0)
Bereavement	17 (5.6)
Mild depression	1 (0.3)
Moderate depression	15 (5.0)
Suicide risk	41 (13.5)
Bipolar disorder	28 (9.2)
Psychotic features	96 (31.7)
Alcohol/drug abuse	14 (4.6)

^a^There were 303 clinical conditions in total for 242 patients, as multiple conditions for the same patient were allowed based on symptoms.

### Depression, Suicide Risk, and Anxiety

Table 3 reports data for 843 adults only. Among them, moderate to severe anxiety or depression scores (≥10) was present in 695 (82.4%), with the remainder (148/843, 17.6%) reporting increased suicide risk (score≥1) despite low to mild depression and anxiety scores. At postintervention, 56 (6.6%) adults had moderate-severe anxiety or depression, and 14 (1.7%) adults had an increased suicide risk. In all, 717 of the 843 (85.1%) adults who were at high risk at baseline were no longer at high risk for CMD at postintervention (ie, PHQ-9 and GAD-7 scores were <10, and the suicide risk score was 0).

Mean depression and anxiety scores reduced significantly postintervention for those individuals identified by ASHAs who had a score ≥10 on the PHQ-9 and/or GAD-7 at the beginning of the intervention. The mean PHQ-9 scores reduced from 13.4 at baseline to 3.1 at 12 months, mean difference −10.3 (95% CI −10.7 to −9.8; *P*<.001; ICC 0.04), and the mean GAD-7 scores reduced from 12.9 at baseline to 1.9 at 12 months, mean difference −11.0 (95% CI −11.4 to −10.6; *P*<.001; ICC 0.08).

**Table 3:** Scores on anxiety (7-item Generalized Anxiety Disorder) and depression scales (9-item Patient Health Questionnaire) for those screened positive by Accredited Social Health Activists and then reinterviewed at postintervention.

Clinical condition	Baseline (n=843), n (%)	Postintervention (n=843), n (%)
Anxiety (percentage with GAD-7^a^≥10)	408 (48.4)	29 (3.4)
Depression (percentage with PHQ-9^b^≥10)	492 (58.4)	55 (6.5)
Anxiety or depression (percentage with GAD-7/PHQ-9≥10)^c^	695 (82.4)	56 (6.6)
Increased self-harm risk (score≥1, with GAD-7 and PHQ-9 scores<10)	148 (17.6)	14 (1.7)

^a^GAD-7: 7-item Generalized Anxiety Disorder.

^b^PHQ-9: 9-item Patient Health Questionnaire.

^c^205 and 21 adults had both GAD-7 and PHQ-9 ≥10 at baseline and postintervention, respectively.

## Discussion

### Principal Findings

In this quasi-experimental study, the use of primary care services for mental health problems increased from 3.3% (30/900) to 81.2% (730/900), following a complex, multifaceted, technology-enabled intervention. The depression and anxiety scores among those who were screened positive for CMDs by nonphysician community health care workers were significantly lower following the intervention.

#### Limitations

There were a number of limitations in this study. First, this is a pre-post design with no controls; hence, the results can only be interpreted as exploratory. Second, the changes in depression and anxiety scores should be interpreted in light of other work that suggests over a 1-year period 50% individuals with CMD could experience natural remission [26]. Although the effect sizes reported in this study were far greater than this, it is reasonable to assume that some proportion of the improvement can be attributed to natural remission. Third, the interrater reliability between the interviewer and ASHA screening was low. It is difficult to comment on the specific reasons for this because of the time lag between the different interviews. This may be partly explained by natural remission, as the period in the 2 screenings was almost 2 months, and natural remission could be as much as 20% in 2 months [26]. Another explanation is the *retest effect* where results from psychiatric research show that retesting using the same instrument can lead to attenuated results because of a number of reasons [27]. Fourth, although this study has measures for symptom assessment, it did not have any measure for functional ability, and future studies may consider having that measure.

#### Common Mental Disorders in the Community

Compared with screen-negative individuals, the screen-positive individuals were older and represented by more women, a higher proportion of individuals with no schooling or who were jobless, and a higher proportion who were separated/widowed/divorced. These findings were similar to extant literature from India and abroad [28,29]. The prevalence of CMD (4.1%) in the community was similar to our earlier study [30] but was substantially lower than national estimates of 10% [2]. One reason could be that alcohol and substance use disorders was not included in our definition of CMD. There was also a time lag between ASHA screening and doctor diagnosis, as individuals visited the doctor as per their convenience. Natural remission could, therefore, contribute to the finding that only one-third of the screen-positive individuals received a clinical diagnosis. However, another reason for fewer screen-positive individuals receiving a clinical diagnosis could be many individuals being hesitant to discuss mental health problems with doctors in the first visit, which is often seen in clinical psychiatry practice. It is also important to note that the criteria which ASHAs and doctors used to define a mental illness were different from the former group using PHQ-9 and GAD-7 scores and the latter group using clinical criteria defined in the mh-GAP toolkit. Most trials use measures such as PHQ-9 and GAD-7 scores, and the reduction in scores in our study was similar for screen-positive people regardless of whether they were clinically diagnosed by the doctor as having a mental illness. This study assesses the individuals at baseline and at postintervention. It may be possible that some individuals may have recurrent depression, and the final score in such cases may not be related to the baseline depressive episode. However, for those individuals who received care from the PHC doctor, we have records of their clinical assessment and follow-up and did not come across any such case.

More than 30% of those clinically assessed had features suggestive of psychosis and were immediately referred to a mental health professional. Psychosis was a difficult symptom for the primary care doctors to identify, and it is possible that some of those may have been false-positives. However, given the limited resources available at PHCs, it was prudent to send any doubtful case to a specialist. Future projects could possibly minimize this by having more formal specialist supervision of primary care doctors, and the same are being planned for the scale up of this project. Another implication of this is that besides the initial training, the doctors could have benefited from a few booster trainings.

#### Mental Health Services Delivery Using Technology

The primary outcome—use of mental health services—increased significantly and was higher than that reported in the Vidarbha Stress and Health Program (VISHRAM) [31]. Unlike our intervention, VISHRAM did not use decision support but did include a referral process to psychiatrists.

In addition, in our project, doctors held *health camps* in villages, and these contributed significantly to the increased use of services. However, both these projects underline the value of providing mental health care through primary health care workers and the ability of such workers to bring about an increase in services uptake. Neither VISHRAM nor SMART Mental Health was a randomized trial, so more robust studies are needed in the future to provide reliable estimates of effectiveness as well as information on cost-effectiveness.

Task shifting has been found to be useful for increasing access to health services in hard to reach communities with few mental health professionals. However, a more detailed understanding about cost-effectiveness is lacking, as are data from LMICs [11,32]. Our earlier research involving a smaller population and shorter intervention period had found task sharing as acceptable, feasible, and effective [30,33]. The lessons learned from this can be applicable to similar settings where the use of technology is possible, where government support to involve the primary health care system is available, and where training and task sharing can be implemented. However, we plan to conduct more robust studies in the future to enhance the impact of the intervention and make it scalable across other situations.

#### Policy and Practice Implications

This research is the largest study from an LMIC using a complex intervention, including an antistigma campaign, task sharing, and EDSSs to care for individuals with CMDs at primary care level. The policy implication of this study is contingent on demonstration scalability, such that such interventions could help realize the objective of the Mental Health Action Plan [34] and National Mental Health Policy [35], which advocates the delivery of mental health care through primary health workers. Interventions such as SMART Mental Health could lead to more accessible and equitable mental health services, with the technology, task sharing, and antistigma components addressing both demand and supply barriers. Thornicroft et al [36] reported that only 1 in 27 individuals with major depression in LMICs received minimally adequate treatment, hence making it more imperative to find disruptive strategies to bridge that gap in LMIC settings. Practicing psychiatrists can help support mental health services delivery in primary care settings by using lessons from this project. This is relevant in both LMICs with limited resources as well as in areas within high-income countries where psychiatrists are limited. Psychiatrists can play a role in training primary care doctors using technology, monitor them, and provide specialist care when needed.

### Conclusions

In conclusion, the technology-enabled mental health services delivery intervention led to a significant increase in uptake of mental health services in the community and improvement in depression and anxiety symptoms. Future studies should use more robust designs so that the results can inform scalable programs for India and potentially other resource-poor settings.

## Appendix

Multimedia Appendix 1 Statistical analysis plan.

Multimedia Appendix 2 Supplementary analyses.

## Abbreviations

ASHA: Accredited Social Health Activist
CMD: common mental disorder
EDSS: electronic decision support system
GAD: Generalized Anxiety Disorder
ICC: intraclass correlation coefficient
LMIC: low- and middle-income country
mhGAP-IG: Mental Health Gap Intervention Guide
OR: odds ratio
PHC: primary health care center
PHQ: Patient Health Questionnaire
SMART: Systematic Medical Appraisal Referral and Treatment
VISHRAM: Vidarbha Stress and Health Program
