# Supplementary material for: The Systematic Medical Appraisal Referral and Treatment Mental Health Project: Quasi-Experimental Study to Evaluate a Technology-Enabled Mental Health Services Delivery Model Implemented in Rural India
Source: J Med Internet Res. 2020 Feb 27;22(2):e15553. doi: 10.2196/15553 (PMC7068463; doi:10.2196/15553)
Supplement: Multimedia Appendix 1 [file jmir_v22i2e15553_app1.pdf]

SMART Mental Health

Innovative provision of primary mental health care in India: The Systematic Medical Appraisal, Referral and Treatment (SMART) Mental Health Programme

Statistical Methodology- SAP and Shells

Author: Anadya Prakash Tripathi, Statistician

Document status: Final

Release date: 24-11-2017

Number of pages: 26

Table of contents: **Page**

Smart mental health Visit schedule and analysis for visit	4
Data selection criteria for Analysis	4
Smart mental health Final Statistical Analysis plan	4
Primary Analysis	4
Case control Analysis of Primary objective	5
Secondary Analysis	5
Depression and Anxiety Score	5
Demographic and Socioeconomic characteristics	5
Statistical Analysis of KAB and BACE	5
Stressful events	6
Sharing personal problems	6
Assessment of Alcohol and substance use	6
Past history of illness	6
Subject referral by ASHA and PHC doctors	6

Smart mental health Mock shells tables

Table 1: Demographic and socioeconomic Characteristics	7
Table 2: Summary for KAB	8
Table 3: Mean scores and change in Mean scores of KAB Questions	14
Table 4: Summary for BACE	15
Table 5: Mean scores and Change in Mean scores for each barrier in the BACE	19
Table 6: Percentage reporting and ranks for each barrier in the BACE ASHA screen positive	20
Table 7a: Summary of screening Status and severity of anxiety and depression	21
Table 7b: Summary of Anxiety and Depression Score of ASHA screen positive	21
Table 7c: Summary of Patients who are Screened Positive by ASHA	22
Table 8a: Change in Depression/Anxiety Score from Baseline to Post Intervention in screen positive	23
Table 8b: Change in Depression/Anxiety Score from Baseline to Post Intervention for those who had a score >=10 at the beginning of the intervention as screened by ASHAs	23

Table 9a: Summary of stressful question for the Patients who are Screened Positive by ASHA 24

Table 9b: Summary of stressful events among the Patients who have Depression or Anxiety Score >=10 24

Table 10: Summary of Stressful Events for the Patients who are Screened Positive by ASHA 25

Table 11: Summary of Sharing Personal Problems for the Patients who are screened Positive by ASHA 25

Table 12: Summary of Assessment of Alcohol and Substance Use for the ASHA screened positive Patients 26

Table 13: Summary of Past History of the ASHA screened positive Patients 27

Table 14a: Summary of treatment History among ASHA screened positive Patients 28

Table 14b: Summary of treatment History during intervention among ASHA screened positive Patients 28

Table 14c: Summary of treatment compliance among treated patients of ASHA screened 28

Table 15: Summary of for Utilization of Health Services for Screen Positive subjects 29

Table 16a: Case control analysis of Change in Depression/Anxiety Score from Baseline to Post Intervention for those who had a score >=10 at the beginning of the intervention 30

Table 16b: Case control analysis for health service utilization 31

Table 17: Subject Referrals during ASHA screening 32

Smart mental health Visit schedule and analysis for visit:

The study will be reported in mainly 2 category:-

1. Baseline (As applicable, this will includes ASHA screening data available in in ASHA Screening 1 and ASHA follow up 1. OR Baseline data collected by study interviewers)
2. Post Intervention (collected by study interviewers)

All the subject at post intervention are screen positive subjects. They were screened positive either at Baseline by study interviewers or at Intervention visits (ASHA screening 1 and ASHA follow-up 1) by ASHA. All the screen positive subjects were followed by ASHAs which is available in the ASHA follow up data.

Unless otherwise stated, the summary tables will be presented under 2 category:-

1. Baseline
2. Post Intervention

The difference between Baseline and Post Intervention will provide effect of intervention.

Apart from the Post Intervention data, post stigma data was also collected. At the time of creating derived data for post Intervention, both data (Post Intervention and post stigma) are appended together and created a single data set. A flag variable containing record ("Post Intervention" & "post stigma") was created was created in derived data set to identify such records.

Unless otherwise stated, all the analysis will be presented for all the subject who are screen positive by ASHA.

Data selection criteria for Analysis:

Unless otherwise stated, for baseline we have to use ASHA screening (ASHA screening 1 and ASHA follow-up 1) data. At post intervention we have to use Post intervention data collected by study interviewers linked with patient ID of ASHA screen positive data.

For certain tables the information at baseline may not be collected by ASHA during their screening. For all such instance, Baseline data collected by study interviewers will be used linked with patient ID of ASHA screen positive data.

Smart mental health Final Statistical Analysis plan Primary Analysis:

The primary objective of the study is:-

To see the difference in anxiety and depression score and health service utilization among the mental health screen positive subject post intervention.

The screen positive subjects at baseline will be looked for their past history for mental health service utilization. The subjects who responded "Yes" against question number 56 "Did you get treated for that mental disorder?" are proportion of subject at baseline who used mental health service.

At post intervention, the number of subjects used health service can be taken from Doctors follow-up data.

The count and percentage will be provided for the subjects who are screen positive for utilization of health services. Proportion of mental health service utilization at baseline and post intervention among the screen positive subject will be statistically analyze by McNemar test. The P-value for comparison of will be presented in same table.

A separate table will be provided for subjects screen positive subjects who actually visited doctor for their treatment at time of intervention. The count and percent by visit will be provided for this.

Case control Analysis of Primary objective:

All the subjects screened positive by ASHA are case here. The subjects who mental health are screen negative by ASHA, but screen positive by interviewer are kind of control in this study setup. Such subjects will be called as control.

A case control analysis will be performed to assess the difference in anxiety and depression score and mental health service utilization.

For this difference in anxiety and depression score for individual subjects will be computed from ASHA screen data and post intervention data. Further this difference in case and control will be statistically analyses by 2 independent sample t-test. Difference of difference in score between case and control along with p-value will be presented.

For health service utilization between cases and control, Count along with percent and Chi- square P-value will be presented.

Secondary Analysis Depression and Anxiety Score:

The subjects are classified as screen positive if he/she fulfil either one of below mentioned three category:-

1. Depression Sore ≥10
2. Anxiety Score ≥10
3. Suicidal tendency Score ≥1

The depression and anxiety will be categorized as Normal if their total score is in between 0-4. The score in between 5-9 will be classify as Mild. The score in between 10-14 will be classify as moderated. The score of 15 or greater will be treated as severe.

The Anxiety and Depression score will be categorized by their severity. The count and percentage for depression and Anxiety at baseline and post baseline will be presented by severity. A separate table will be presented for depression score ≥10 and anxiety score ≥10 at baseline and post intervention.

A separated table will be provides for the only screen positive subjects with the count and percentage of the symptoms which lead them to be screen positive.

The summary statistics (N, Mean, STD, Min and Max) for anxiety and depression score by visit will be presented. The difference in score between baseline and post intervention will be summarized in similar fashion. The mean difference in score between baseline and post intervention will be statistically analyzed by paired t-test. The p-value from the analysis will be presented in same table.

Demographic and Socioeconomic characteristics:

The count and frequency for the Social characteristic like Gender, occupation, education and marital status will be presented. Age will be summarized by mean SD and range. The occupation and education was not collected at post intervention. Hence the record from baseline will be taken to post intervention for occupation and education.

Statistical Analysis of KAB and BACE:

For every question related to KAB and BACE, the count and percentage will be presented for every response for the concern question.

Further to that for every question, mean score and SD along with number of people responded to different questions of KAB and BACE will be presented. The difference in score between baseline and post intervention will also be analyses by t-test. The mean SD and the number of people responded at both visit along with p-value will be presented.

For KAB the Total Behaviors SCORE (Except First Question) and For BACE, the Total BACE Score, will also be analyzed and summarized in similar fashion.

A separate table for BACE will be presented for % reporting barrier to any degree and % reporting as a major barrier along with their rank for highest proportion rating as a major barrier. The difference in proportion for %reported a major barrier will be analyses by two- proportion z-test and corresponding p-value will pre presented.

Stressful events:

The count and percentage will be provided for the screen positive subjects for the questions of stressful events for which they responded as yes at baseline.
A separate table will provided for the questions of stressful events for which they responded as yes for the subjects who had Depression or Anxiety Score >=10.
The stressful events will be summarized in 4 category (0, 1, 2-3, ≥4) by count and percentage. Also Descriptive Statistics (n, mean, SD, Min, and Max) for the patients who have total score greater than 1 will be provided.

Sharing personal problems:

The count and percentage will be provided for those subjects who had responded yes for the Question "When you have a personal problem do you share it with anyone?" at baseline.

Assessment of Alcohol and substance use:

The count and percentage will be provided for those subjects who had responded yes for all the 10 different question of alcohol and substance use section.

Past history of illness:

The count and percentage will be provided for those subjects who had responded yes for the questions of heart attack or angina or stroke or diabetes or cancer.

The count and percentage will be provided separately for those subjects who had responded yes for the questions of mental disorder with count and percentage for those subjects who had responded yes for the questions "Did you get treated for that mental disorder?" in similar fashion.

Subject referral by ASHA and PHC doctors:

The count and percentage will be provided for those subjects who were referred by ASHA to PHC doctor during intervention and among all such screen positive subjects again count and percentage will be provided for those subjects who were referred by PHC doctor for more specialized care.

Table1: Demographic and Socioeconomic characteristics

Characteristics	Baseline (N=XX) n%	Post Intervention (N=XX) n%
**Gender**		
Female	**xx(xx.x%)**	**xx(xx.x%)**
Male	**xx(xx.x%)**	**xx(xx.x%)**
**Occupation**		
Housewife/Retired	**xx(xx.x%)**	**xx(xx.x%)**
Organized Sector	**xx(xx.x%)**	**xx(xx.x%)**
Other	**xx(xx.x%)**	**xx(xx.x%)**
Unorganized Sector	**xx(xx.x%)**	**xx(xx.x%)**
**Education**		
Graduate/Postgraduate	**xx(xx.x%)**	**xx(xx.x%)**
High School	**xx(xx.x%)**	**xx(xx.x%)**
No School	**xx(xx.x%)**	**xx(xx.x%)**
Other	**xx(xx.x%)**	**xx(xx.x%)**
Primary School	**xx(xx.x%)**	**xx(xx.x%)**
**Marital Status**		
Currently Married	**xx(xx.x%)**	**xx(xx.x%)**
Never Married	**xx(xx.x%)**	**xx(xx.x%)**
Separated/Divorced/Widowed	**xx(xx.x%)**	**xx(xx.x%)**
**Age(Years)**		
Mean(SD)	**xx.xx(xx.xxx)**	**xx.xx(xx.xxx)**
Range	**xx.x; xx.x**	**xx.x; xx.x**

N=Total no. of patients in that visit. n= No. of patients with particular characteristic.

Table 2: Summary for KAB

	Question	Baseline (N=XX) n%	Post Intervention (N=XX) n%
**Context***	**Have you seen or heard any information about mental health or mental illness issues in the last year, in any of these ways?**		
	[Newspaper/TV]	**xx(xx.x%)**	**xx(xx.x%)**
	[Other]	**xx(xx.x%)**	**xx(xx.x%)**
	[People talking about it/ Other]	**xx(xx.x%)**	**xx(xx.x%)**
	[People talking about it]	**xx(xx.x%)**	**xx(xx.x%)**
	[TV/Health center/People talking about it / Other]	**xx(xx.x%)**	**xx(xx.x%)**
	[TV/ Other]	**xx(xx.x%)**	**xx(xx.x%)**
	[TV/People talking about it/ Other]	**xx(xx.x%)**	**xx(xx.x%)**
	[TV]	**xx(xx.x%)**	**xx(xx.x%)**
	**Where do people in this community first go to seek care for mental illness?**		
	[Hospital]	**xx(xx.x%)**	**xx(xx.x%)**
	[Local clinic]	**xx(xx.x%)**	**xx(xx.x%)**
	[Neighbor/ Community Member]	**xx(xx.x%)**	**xx(xx.x%)**
	[Nowhere/ Care is not available]	**xx(xx.x%)**	**xx(xx.x%)**
	[Other]	**xx(xx.x%)**	**xx(xx.x%)**
	[Religious or spiritual advisor]	**xx(xx.x%)**	**xx(xx.x%)**
	[Traditional healer]	**xx(xx.x%)**	**xx(xx.x%)**

	**Do any of the following people you know have a mental illness?**		
	[Family member in this household]	**xx(xx.x%)**	**xx(xx.x%)**
	[Family member outside this household]	**xx(xx.x%)**	**xx(xx.x%)**
	[Friends/Acquaintance]	**xx(xx.x%)**	**xx(xx.x%)**
	[Neighbor]	**xx(xx.x%)**	**xx(xx.x%)**
	[No one known]	**xx(xx.x%)**	**xx(xx.x%)**
	[Someone else]	**xx(xx.x%)**	**xx(xx.x%)**
**Knowledge**	**Mentally ill people tend to be violent**		
	Agree Slightly	**xx(xx.x%)**	**xx(xx.x%)**
	Agree Strongly	**xx(xx.x%)**	**xx(xx.x%)**
	Disagree Slightly	**xx(xx.x%)**	**xx(xx.x%)**
	Disagree Strongly	**xx(xx.x%)**	**xx(xx.x%)**
	Don’t know	**xx(xx.x%)**	**xx(xx.x%)**
	Neither agree or disagree	**xx(xx.x%)**	**xx(xx.x%)**
	**People with mental illness can’t live a good rewarding life**		
	Agree Slightly	**xx(xx.x%)**	**xx(xx.x%)**
	Agree Strongly	**xx(xx.x%)**	**xx(xx.x%)**
	Disagree Slightly	**xx(xx.x%)**	**xx(xx.x%)**
	Disagree Strongly	**xx(xx.x%)**	**xx(xx.x%)**
	Don’t know	**xx(xx.x%)**	**xx(xx.x%)**
	Neither agree or disagree	**xx(xx.x%)**	**xx(xx.x%)**

	**People with severe mental problems can fully recover**		
	Agree Slightly	**xx(xx.x%)**	**xx(xx.x%)**
	Agree Strongly	**xx(xx.x%)**	**xx(xx.x%)**
	Disagree Slightly	**xx(xx.x%)**	**xx(xx.x%)**
	Disagree Strongly	**xx(xx.x%)**	**xx(xx.x%)**
	Don’t know	**xx(xx.x%)**	**xx(xx.x%)**
	Neither agree or disagree	**xx(xx.x%)**	**xx(xx.x%)**
	**Medication can be an effective treatment for people with mental health problems**		
	Agree Slightly	**xx(xx.x%)**	**xx(xx.x%)**
	Agree Strongly	**xx(xx.x%)**	**xx(xx.x%)**
	Disagree Slightly	**xx(xx.x%)**	**xx(xx.x%)**
	Disagree Strongly	**xx(xx.x%)**	**xx(xx.x%)**
	Don’t know	**xx(xx.x%)**	**xx(xx.x%)**
	Neither agree or disagree	**xx(xx.x%)**	**xx(xx.x%)**
**Attitude**	**Mentally ill people shouldn’t get married**		
	Agree Slightly	**xx(xx.x%)**	**xx(xx.x%)**
	Agree Strongly	**xx(xx.x%)**	**xx(xx.x%)**
	Disagree Slightly	**xx(xx.x%)**	**xx(xx.x%)**
	Disagree Strongly	**xx(xx.x%)**	**xx(xx.x%)**
	Don’t know	**xx(xx.x%)**	**xx(xx.x%)**
	Neither agree or disagree	**xx(xx.x%)**	**xx(xx.x%)**

People with mental health problems are far less of a danger than most people suppose

Agree Slightly	**xx(xx.x%)**	**xx(xx.x%)**
Agree Strongly	**xx(xx.x%)**	**xx(xx.x%)**
Disagree Slightly	**xx(xx.x%)**	**xx(xx.x%)**
Disagree Strongly	**xx(xx.x%)**	**xx(xx.x%)**
Don’t know	**xx(xx.x%)**	**xx(xx.x%)**
Neither agree or disagree	**xx(xx.x%)**	**xx(xx.x%)**

We need to adopt a far more tolerant attitude toward people with mental illness in our society

Agree Slightly	**xx(xx.x%)**	**xx(xx.x%)**
Agree Strongly	**xx(xx.x%)**	**xx(xx.x%)**
Disagree Slightly	**xx(xx.x%)**	**xx(xx.x%)**
Disagree Strongly	**xx(xx.x%)**	**xx(xx.x%)**
Don’t know	**xx(xx.x%)**	**xx(xx.x%)**
Neither agree or disagree	**xx(xx.x%)**	**xx(xx.x%)**

People with mental health problems shouldn’t be given any responsibility

Agree Slightly	**xx(xx.x%)**	**xx(xx.x%)**
Agree Strongly	**xx(xx.x%)**	**xx(xx.x%)**
Disagree Slightly	**xx(xx.x%)**	**xx(xx.x%)**
Disagree Strongly	**xx(xx.x%)**	**xx(xx.x%)**
Don’t know	**xx(xx.x%)**	**xx(xx.x%)**
Neither agree or disagree	**xx(xx.x%)**	**xx(xx.x%)**

**Behavior**	**I would be willing to live with someone with a mental health problem**		
	Agree Slightly	**xx(xx.x%)**	**xx(xx.x%)**
	Agree Strongly	**xx(xx.x%)**	**xx(xx.x%)**
	Disagree Slightly	**xx(xx.x%)**	**xx(xx.x%)**
	Disagree Strongly	**xx(xx.x%)**	**xx(xx.x%)**
	Don't know	**xx(xx.x%)**	**xx(xx.x%)**
	Neither agree or disagree	**xx(xx.x%)**	**xx(xx.x%)**
	**I would be willing to work with someone with a mental health problem**		
	Agree Slightly	**xx(xx.x%)**	**xx(xx.x%)**
	Agree Strongly	**xx(xx.x%)**	**xx(xx.x%)**
	Disagree Slightly	**xx(xx.x%)**	**xx(xx.x%)**
	Disagree Strongly	**xx(xx.x%)**	**xx(xx.x%)**
	Don't know	**xx(xx.x%)**	**xx(xx.x%)**
	Neither agree or disagree	**xx(xx.x%)**	**xx(xx.x%)**
	**I would be willing to live nearby someone with a mental health problem**		
	Agree Slightly	**xx(xx.x%)**	**xx(xx.x%)**
	Agree Strongly	**xx(xx.x%)**	**xx(xx.x%)**
	Disagree Slightly	**xx(xx.x%)**	**xx(xx.x%)**
	Disagree Strongly	**xx(xx.x%)**	**xx(xx.x%)**
	Don't know	**xx(xx.x%)**	**xx(xx.x%)**
	Neither agree or disagree	**xx(xx.x%)**	**xx(xx.x%)**

**I would be willing to continue a relationship with a friend who developed a mental health problem**		
[Family]	**xx(xx.x%)**	**xx(xx.x%)**
[Friends/ Family]	**xx(xx.x%)**	**xx(xx.x%)**
[Friends]	**xx(xx.x%)**	**xx(xx.x%)**
[No one]	**xx(xx.x%)**	**xx(xx.x%)**

**A subject can be counted more than once for context questions and only top 5 are reported. KAB=Knowledge, Attitude, Behavior.*

Table 3: Mean scores and change in Mean scores of KAB Questions:

Category	Question	Baseline<br>N, Mean(SD)	Post intervention<br>N, Mean(SD)	Post intervention-Baseline<br>N, Mean(SD) P-value
Knowledge	Mentally ill people tend to be violent	**xx, xx.xx(xx.xxx)**	**xx, xx.xx(xx.xxx)**	**xx, xx.xx(xx.xxx) 0.xxxx**
	People with mental illness can't live a good, rewarding life	**xx, xx.xx(xx.xxx)**	**xx, xx.xx(xx.xxx)**	**xx, xx.xx(xx.xxx) 0.xxxx**
	People with severe mental health problems can fully recover	**xx, xx.xx(xx.xxx)**	**xx, xx.xx(xx.xxx)**	**xx, xx.xx(xx.xxx) 0.xxxx**
	Medication can be an effective treatment for people with mental health problems	**xx, xx.xx(xx.xxx)**	**xx, xx.xx(xx.xxx)**	**xx, xx.xx(xx.xxx) 0.xxxx**
Attitude	Mentally ill people shouldn't get married	**xx, xx.xx(xx.xxx)**	**xx, xx.xx(xx.xxx)**	**xx, xx.xx(xx.xxx) 0.xxxx**
	People with mental health problems are far less danger than most people suppose	**xx, xx.xx(xx.xxx)**	**xx, xx.xx(xx.xxx)**	**xx, xx.xx(xx.xxx) 0.xxxx**
	We need to adopt a far more tolerant attitude toward people with mental illness in our society	**xx, xx.xx(xx.xxx)**	**xx, xx.xx(xx.xxx)**	**xx, xx.xx(xx.xxx) 0.xxxx**
	People with mental health problems shouldn't be given any responsibility	**xx, xx.xx(xx.xxx)**	**xx, xx.xx(xx.xxx)**	**xx, xx.xx(xx.xxx) 0.xxxx**
Behavior	I would be willing to live with someone with a mental health problem	**xx, xx.xx(xx.xxx)**	**xx, xx.xx(xx.xxx)**	**xx, xx.xx(xx.xxx) 0.xxxx**
	I would be willing to work with someone with a mental health problem	**xx, xx.xx(xx.xxx)**	**xx, xx.xx(xx.xxx)**	**xx, xx.xx(xx.xxx) 0.xxxx**
	I would be willing to live nearby with someone with a mental health problem	**xx, xx.xx(xx.xxx)**	**xx, xx.xx(xx.xxx)**	**xx, xx.xx(xx.xxx) 0.xxxx**
	I would be willing to continue a relationship with a friend who developed a mental health problem	**xx, xx.xx(xx.xxx)**	**xx, xx.xx(xx.xxx)**	**xx, xx.xx(xx.xxx) 0.xxxx**
	Overall Behaviors SCORE	**xx, xx.xx(xx.xxx)**	**xx, xx.xx(xx.xxx)**	**xx, xx.xx(xx.xxx) 0.xxxx**

**P value is calculated using t-test .KAB=Knowledge, Attitude, Behavior*
For mean of difference, subject who have responded at both visit, have been considered for summary.

Table4: Summary of BAC

Question	Baseline (N=XX) N(%)	Post intervention (N=XX) N(%)
**Concern that I might have seen as weak for having a mental health problem**		
A little	**xx(xx.x%)**	**xx(xx.x%)**
A lot	**xx(xx.x%)**	**xx(xx.x%)**
NA	**xx(xx.x%)**	**xx(xx.x%)**
Not at all	**xx(xx.x%)**	**xx(xx.x%)**
Quite a lot	**xx(xx.x%)**	**xx(xx.x%)**
**Concern that it might harm my chances when applying for jobs**		
A little	**xx(xx.x%)**	**xx(xx.x%)**
A lot	**xx(xx.x%)**	**xx(xx.x%)**
NA	**xx(xx.x%)**	**xx(xx.x%)**
Not at all	**xx(xx.x%)**	**xx(xx.x%)**
Quite a lot	**xx(xx.x%)**	**xx(xx.x%)**
**Concern about what my family might think / say / do or feel**		
A little	**xx(xx.x%)**	**xx(xx.x%)**
A lot	**xx(xx.x%)**	**xx(xx.x%)**
NA	**xx(xx.x%)**	**xx(xx.x%)**
Not at all	**xx(xx.x%)**	**xx(xx.x%)**
Quite a lot	**xx(xx.x%)**	**xx(xx.x%)**

**Feeling embarrassed or ashamed**		
A little	**xx(xx.x%)**	**xx(xx.x%)**
A lot	**xx(xx.x%)**	**xx(xx.x%)**
NA	**xx(xx.x%)**	**xx(xx.x%)**
Not at all	**xx(xx.x%)**	**xx(xx.x%)**
Quite a lot	**xx(xx.x%)**	**xx(xx.x%)**
**Concern that I might be seen as crazy**		
A little	**xx(xx.x%)**	**xx(xx.x%)**
A lot	**xx(xx.x%)**	**xx(xx.x%)**
NA	**xx(xx.x%)**	**xx(xx.x%)**
Not at all	**xx(xx.x%)**	**xx(xx.x%)**
Quite a lot	**xx(xx.x%)**	**xx(xx.x%)**
**Concern that I might be seen as bad parents**		
A little	**xx(xx.x%)**	**xx(xx.x%)**
A lot	**xx(xx.x%)**	**xx(xx.x%)**
NA	**xx(xx.x%)**	**xx(xx.x%)**
Not at all	**xx(xx.x%)**	**xx(xx.x%)**
Quite a lot	**xx(xx.x%)**	**xx(xx.x%)**
**Concern that people I know might find out**		
A little	**xx(xx.x%)**	**xx(xx.x%)**
A lot	**xx(xx.x%)**	**xx(xx.x%)**
NA	**xx(xx.x%)**	**xx(xx.x%)**
Not at all	**xx(xx.x%)**	**xx(xx.x%)**
Quite a lot	**xx(xx.x%)**	**xx(xx.x%)**

**Concern that people might not take me seriously if they found out I was having professional care**		
A little	**xx(xx.x%)**	**xx(xx.x%)**
A lot	**xx(xx.x%)**	**xx(xx.x%)**
NA	**xx(xx.x%)**	**xx(xx.x%)**
Not at all	**xx(xx.x%)**	**xx(xx.x%)**
Quite a lot	**xx(xx.x%)**	**xx(xx.x%)**
**Not wanting a mental health problem to be on my medical records**		
A little	**xx(xx.x%)**	**xx(xx.x%)**
A lot	**xx(xx.x%)**	**xx(xx.x%)**
NA	**xx(xx.x%)**	**xx(xx.x%)**
Not at all	**xx(xx.x%)**	**xx(xx.x%)**
Quite a lot	**xx(xx.x%)**	**xx(xx.x%)**
**Concern that my children may be taken in to care or that I may lose access or custody without my consent**		
A little	**xx(xx.x%)**	**xx(xx.x%)**
A lot	**xx(xx.x%)**	**xx(xx.x%)**
NA	**xx(xx.x%)**	**xx(xx.x%)**
Not at all	**xx(xx.x%)**	**xx(xx.x%)**
Quite a lot	**xx(xx.x%)**	**xx(xx.x%)**
**Concern that what my friends might think/ say or do**		
A little	**xx(xx.x%)**	**xx(xx.x%)**
A lot	**xx(xx.x%)**	**xx(xx.x%)**
NA	**xx(xx.x%)**	**xx(xx.x%)**
Not at all	**xx(xx.x%)**	**xx(xx.x%)**
Quite a lot	**xx(xx.x%)**	**xx(xx.x%)**

**Concern about what people at work might think/ say or do**		
A little	**xx(xx.x%)**	**xx(xx.x%)**
A lot	**xx(xx.x%)**	**xx(xx.x%)**
NA	**xx(xx.x%)**	**xx(xx.x%)**
Not at all	**xx(xx.x%)**	**xx(xx.x%)**
Quite a lot	**xx(xx.x%)**	**xx(xx.x%)**

Table 5: Mean Scores and Change in mean scores for each barrier in the BACE

Question	Baseline<br>N, Mean(SD)	Post intervention<br>N, Mean(SD)	Post intervention-Baseline<br>N, Mean(SD) P-value
Concern that I might be seen as weak for having a mental health problem	**xx, xx.xx(xx.xxx)**	**xx, xx.xx(xx.xxx)**	**xx, xx.xx(xx.xxx) 0.xxxx**
Concern that it might harm my chances when applying for jobs	**xx, xx.xx(xx.xxx)**	**xx, xx.xx(xx.xxx)**	**xx, xx.xx(xx.xxx) 0.xxxx**
Concern about what my family think, say do or feel	**xx, xx.xx(xx.xxx)**	**xx, xx.xx(xx.xxx)**	**xx, xx.xx(xx.xxx) 0.xxxx**
Feeling embarrassed or ashamed	**xx, xx.xx(xx.xxx)**	**xx, xx.xx(xx.xxx)**	**xx, xx.xx(xx.xxx) 0.xxxx**
Concern that I might be seen as ‘crazy’	**xx, xx.xx(xx.xxx)**	**xx, xx.xx(xx.xxx)**	**xx, xx.xx(xx.xxx) 0.xxxx**
Concern that I might be seen as bad parents	**xx, xx.xx(xx.xxx)**	**xx, xx.xx(xx.xxx)**	**xx, xx.xx(xx.xxx) 0.xxxx**
Concern that people I know might find out	**xx, xx.xx(xx.xxx)**	**xx, xx.xx(xx.xxx)**	**xx, xx.xx(xx.xxx) 0.xxxx**
Concern that people might not take me seriously if they found out I was having professional care	**xx, xx.xx(xx.xxx)**	**xx, xx.xx(xx.xxx)**	**xx, xx.xx(xx.xxx) 0.xxxx**
Not wanting a mental health problem to be on my medical records	**xx, xx.xx(xx.xxx)**	**xx, xx.xx(xx.xxx)**	**xx, xx.xx(xx.xxx) 0.xxxx**
Concern that my children may be taken in to care or that I may lose access or custody without my consent	**xx, xx.xx(xx.xxx)**	**xx, xx.xx(xx.xxx)**	**xx, xx.xx(xx.xxx) 0.xxxx**
Concern about what my friends might think, say or do	**xx, xx.xx(xx.xxx)**	**xx, xx.xx(xx.xxx)**	**xx, xx.xx(xx.xxx) 0.xxxx**
Concern about what people at work might think, say or do	**xx, xx.xx(xx.xxx)**	**xx, xx.xx(xx.xxx)**	**xx, xx.xx(xx.xxx) 0.xxxx**
Overall BACE score	**xx, xx.xx(xx.xxx)**	**xx, xx.xx(xx.xxx)**	**xx, xx.xx(xx.xxx) 0.xxxx**

**P value is calculated using t-test. BACE=barrier to access care evaluation-Treatment Stigma Subscale. For mean of difference, Subject who have responded to both the visit, have been considered for summary.*

Table 6: Percentage reporting and ranks for each barrier in the BACE ASHA screen positive

	----------------Baseline---------------			----------Post Intervention-----------			
**Question**	**%reporting barrier to any degree**	**%reporting as major barrier (a lot)**	***** Rank**	**%reporting barrier to any degree**	**%reporting as major barrier (a lot)**	***** Rank**	*** P-Value Visit 2-3**
Concern that I might be seen as weak for having a mental health problem	xx.xx	xx.xx	x	xx.xx	xx.xx	x	0.xxx
Concern that it might harm my chances when applying for jobs	xx.xx	xx.xx	x	xx.xx	xx.xx	x	0.xxx
Concern about what my family might think, say, do or feel	xx.xx	xx.xx	x	xx.xx	xx.xx	x	0.xxx
Feeling embarrassed or ashamed	xx.xx	xx.xx	x	xx.xx	xx.xx	x	0.xxx
Concern that I might be seen as crazy	xx.xx	xx.xx	x	xx.xx	xx.xx	x	0.xxx
Concern that I might be seen as a bad parent	xx.xx	xx.xx	x	xx.xx	xx.xx	x	0.xxx
Concern that people I know might find out	xx.xx	xx.xx	x	xx.xx	xx.xx	x	0.xxx
Concern that people might not take me seriously if they found out I was having professional care	xx.xx	xx.xx	x	xx.xx	xx.xx	x	0.xxx
Not wanting a mental health problem to be on my medical records	xx.xx	xx.xx	x	xx.xx	xx.xx	x	0.xxx
Concern that my children may be taken into care or that I may lose access or custody without my consent	xx.xx	xx.xx	x	xx.xx	xx.xx	x	0.xxx
Concern about what my friends might think, say or do	xx.xx	xx.xx	x	xx.xx	xx.xx	x	0.xxx
Concern about what people at work might think, say or do	xx.xx	xx.xx	x	xx.xx	xx.xx	x	0.xxx
Overall Mean	xx.xx	xx.xx	x	xx.xx	xx.xx	x	0.xxx

** P-value calculated based on difference in proportion reported as major barrier.*

**** Rank (1=item has highest proportion rating as a major barrier)*

Table 7a. Summary of screening Status and severity of anxiety and depression

Screened by	Visit	---Screening Status---			----------Anxiety----------			-------Depression--------		
		N	Positives n (%)	Negatives n (%)	Mild 5-9	Moderate 10-14	Severe >=15	Mild 5-9	Moderate 10-14	Severe >=15
ASHA	Baseline	xxxxx	xxxx (xx.xx)	xxxx (xx.xx)	xxx (xx.xx)	xxx (xx.xx)	xxx (xx.xx)	xxx (xx.xx)	xxx (xx.xx)	xxx (xx.xx)
	Post Intervention	xxxxx	xxxx (xx.xx)	xxxx (xx.xx)	xxx (xx.xx)	xxx (xx.xx)	xxx (xx.xx)	xxx (xx.xx)	xxx (xx.xx)	xxx (xx.xx)
Interviewer	Baseline	xxxxx	xxxx (xx.xx)	xxxx (xx.xx)	xxx (xx.xx)	xxx (xx.xx)	xxx (xx.xx)	xxx (xx.xx)	xxx (xx.xx)	xxx (xx.xx)
	Post Intervention	xxxxx	xxxx (xx.xx)	xxxx (xx.xx)	xxx (xx.xx)	xxx (xx.xx)	xxx (xx.xx)	xxx (xx.xx)	xxx (xx.xx)	xxx (xx.xx)

Table 7b: Summary of Anxiety and Depression Score of ASHA screen positive

Category	Severity	Total Score	Baseline (N=xx) n (%)	Post intervention (N=XX) n (%)
Anxiety	Normal	0-4	xxxx(xx.xx)	xxxx(xx.xx)
	Mild	5-9	xxxx(xx.xx)	xxxx(xx.xx)
	Moderate	10-14	xxxx(xx.xx)	xxxx(xx.xx)
	Severe	>=15	xxxx(xx.xx)	xxxx(xx.xx)
Depression	Normal	0-4	xxxx(xx.xx)	xxxx(xx.xx)
	Mild	5-9	xxxx(xx.xx)	xxxx(xx.xx)
	Moderate	10-14	xxxx(xx.xx)	xxxx(xx.xx)
	Severe	>=15	xxxx(xx.xx)	xxxx(xx.xx)

N=Total no of patients in particular visit. n=No. of patients in particular category Patient is counted only once in either category.

Table 7c: Summary of Patients who are Screened positive by ASHAs.

	Baseline(N=xxxxx) N(%)	Post Intervention(N=xxxxx) N(%)
Depression Score >=10	xxx (xx.xx)	xxx (xx.xx)
Anxiety Score >=10	xxx (xx.xx)	xxx (xx.xx)
Suicide risk Score >=1	xxx (xx.xx)	xxx (xx.xx)
Anxiety or Depression Score >=10	xxx (xx.xx)	xxx (xx.xx)

N=Total no. of patients who are screened positives. Percentages are calculated based on patients who were screened positive.

Table 8a: Change in Depression/Anxiety Score from Baseline to Post Intervention in screen positive

		Anxiety					Depression				
**Screened by**	**Visit**	**N**	**Mean (SD)**	**Midian**	**Min, Max**	**P-value**	**N**	**Mean (SD)**	**Midian**	**Min, Max**	**P-value**
ASHA	Baseline	xxxxx	xx.xx (x.xxx)	xx.xx	xx.x, xx.x		xxxxx	xx.xx (x.xxx)	xx.xx	xx.x, xx.x	
	Post Intervention	xxxxx	xx.xx (x.xxx)	xx.xx	xx.x, xx.x		xxxxx	xx.xx (x.xxx)	xx.xx	xx.x, xx.x	
	Difference	xxxxx	xx.xx (x.xxx)	xx.xx	xx.x, xx.x	0.xxx	xxxxx	xx.xx (x.xxx)	xx.xx	xx.x, xx.x	0.xxx
Interviewer	Baseline	xxxxx	xx.xx (x.xxx)	xx.xx	xx.x, xx.x		xxxxx	xx.xx (x.xxx)	xx.xx	xx.x, xx.x	
	Post Intervention	xxxxx	xx.xx (x.xxx)	xx.xx	xx.x, xx.x		xxxxx	xx.xx (x.xxx)	xx.xx	xx.x, xx.x	
	Difference	xxxxx	xx.xx (x.xxx)	xx.xx	xx.x, xx.x	0.xxx	xxxxx	xx.xx (x.xxx)	xx.xx	xx.x, xx.x	0.xxx

Table 8b: Change in Depression/Anxiety Score from Baseline to Post Intervention for those who had a score >=10 at the beginning of the intervention as screened by ASHAs

	Anxiety					Depression				
**Visit**	**N**	**Mean (SD)**	**Midian**	**Min, Max**	**P-value**	**N**	**Mean (SD)**	**Midian**	**Min, Max**	**P-value**
Baseline	xxxxx	xx.xx (x.xxx)	xx.xx	xx.x, xx.x		xxxxx	xx.xx (x.xxx)	xx.xx	xx.x, xx.x	
Post Intervention	xxxxx	xx.xx (x.xxx)	xx.xx	xx.x, xx.x		xxxxx	xx.xx (x.xxx)	xx.xx	xx.x, xx.x	
Difference	xxxxx	xx.xx (x.xxx)	xx.xx	xx.x, xx.x	0.xxxx	xxxxx	xx.xx (x.xxx)	xx.xx	xx.x, xx.x	0.xxxx

Table 9a: Summary of stressful question for the Patients who are Screened Positive by ASHA

**Questions**	**N=(xx) n(%)**
Did you get married in the last 1 year?	xxx (xx.xx)
Did you get separated/divorced in the last 1 year?	xxx (xx.xx)
Did your spouse die in the last 1 year?	xxx (xx.xx)
Did any of your loved ones die in the last 1 year?	xxx (xx.xx)
Did you have a baby in the last 1 year?	xxx (xx.xx)
Did you lose your job in the last 1 year?	xxx (xx.xx)
Did you retire in the last 1 year?	xxx (xx.xx)
Did you or your loved one suffer any major illness/injury in the last 1 year?	xxx (xx.xx)
Did you have any problems with your boyfriend/girlfriend in the last 1 year?	xxx (xx.xx)
Did you have any major problems with your school/college performance in the last 1 year?	xxx (xx.xx)
Did you have any major financial problems in the last year?	xxx (xx.xx)
Did you face any natural disaster or stolen livestock or death of livestock, or crop failure or forced migration leading to loss of income or property?	xxx (xx.xx)
Did you experience any major crime or were a victim of a major crime like robbery, assault/beating, murder/attempted murder, sexual violence?	xxx (xx.xx)

N=Total no. of screen positive patients. n=No. of patients in particular category

Table 9b: Summary of stressful events among the Patients who have Depression or Anxiety Score >=10

The shell of table 10b will be exact similar to Table 10a.

Table 10: Summary of Stressful Events for the Patients who are Screened Positive by ASHA

Number of Stressful events	**(N=xxxx)**<br>**n(%)**
0	xxx (xx.xx)
1	xxx (xx.xx)
2-3	xxx (xx.xx)
>=4	xxx (xx.xx)
Descriptive Statistics (Patients who have total score greater than 1)	
N	xxxx
Mean (SD)	x.xx (x.xxx)
Median	x.xx
Min, Max	x, x

N=Total no. of patients in Baseline; n=No. of patients in particular category

Table 11: Summary of Sharing Personal Problems for the Patients who are Screened Positive by ASHA

	**(N=xxxx)**<br>**n(%)**
When you have a personal problem do you share it with anyone?	**xx(xx.xx%)**

N=Total no. of patients in Baseline; n=No. of patients in particular category

Table 12: Summary of Assessment of Alcohol and Substance Use for the ASHA screened positive Patients

**Question**	**Baseline (N=xxxxx) n (%)**	**Post Intervention (N=xxxxx) n (%)**
Tobacco products (cigarettes, bidi, gutka, cigars, etc.)	xxx (xx.xx)	xxx (xx.xx)
Alcoholic beverages (beer, wine, spirits, etc.)	xxx (xx.xx)	xxx (xx.xx)
Cannabis (marijuana, pot, grass, hash, etc.)	xxx (xx.xx)	xxx (xx.xx)
Cocaine (coke, crack, etc.)	xxx (xx.xx)	xxx (xx.xx)
Opioids (heroin, morphine, methadone, codeine, etc.)	xxx (xx.xx)	xxx (xx.xx)
Amphetamine type stimulants (speed, diet pills, ecstasy,	xxx (xx.xx)	xxx (xx.xx)
Inhalants (nitrous, glue, petrol, paint thinner, etc.)	xxx (xx.xx)	xxx (xx.xx)
Sedatives or Sleeping Pills (Valium, Serepax, Rohypnol,	xxx (xx.xx)	xxx (xx.xx)
Hallucinogens (LSD, acid, mushrooms, PCP, Special K, etc.)	xxx (xx.xx)	xxx (xx.xx)
Other - specify:	xxx (xx.xx)	xxx (xx.xx)

N=Total no. of patients in Baseline; n=No. of patients in particular category

Table 13: Summary of Past History of the ASHA screened positive Patients

**Question**	**Baseline (N=xxxxx) n (%)**
Have you ever been told by a health provider that you have heart attack or angina?	(xx.xx) xxx
Have you ever been told by a doctor that you have or had Stroke?	(xx.xx) xxx
Have you ever been told by a doctor that you have or have had Diabetes?	(xx.xx) xxx
Have you ever been told by a doctor that you have or have had Cancer?	(xx.xx) xxx
Have you ever been diagnosed to have any mental disorder?	(xx.xx) xxx
Did you get treated for that mental disorder?	(xx.xx) xxx

N=Total no. of patients in Baseline; n=No. of patients in particular category
Percentages for (Did you get treated for that mental disorder?) Is calculated on number of patients diagnosed for mental disorder.

Table 14a: Summary of treatment History among ASHA screened positive Patients

Question	Baseline(N=xxxx) n(%)
Are you currently taking any western medicines regularly?	xx(xx.xx)
Are you currently taking any medicines for mental disorders regularly?	xx(xx.xx)

N=Total no. of patients in Baseline; n=No. of patients in particular category

Table 14b: Summary of treatment History during intervention among ASHA screened positive Patients

Question	Baseline(N=xxxx) n(%)
Did you get treated for your mental health condition as indicated by ASHA/Interviewer	xx(xx.xx)

N=Total no. of patients in Baseline; n=No. of patients in particular category

Table 14c: Summary of treatment compliance among treated patients of ASHA screened positive

Question	Baseline(N=xxxx) n(%)
Did you take medicines as prescribed by the primary care doctor on a regular basis?	xx(xx.xx)

N=Total no. of patients in Baseline; n=No. of patients in particular category

Table 15: Summary of for Utilization of Health Services for Screen Positive subjects

**Screen Positive**	**Health Service used ****	**Baseline (N=xxxxx) n (%)**	**Post Intervention (N=xxxxx) n (%)**	**P-value**
By ASHA	Yes	xxx (xx.xx)	xxx (xx.xx)	0.xxxx
	No	xxx (xx.xx)	xxx (xx.xx)	
By Interviewer*	No	xxx (xx.xx)	xxx (xx.xx)	0.xxxx
	Yes	xxx (xx.xx)	xxx (xx.xx)	

** This includes only those patients who were screen positive by Study Interviewer but not by ASHA.*
*** Health Service used: At baseline: Response from the question "Did you get treated for that mental disorder?"*
At Post Intervention: Response from the question "Did you get treated for your mental health condition as indicated by ASHA/Interviewer?"
P-Value is calculated based on Mc-nimar test for matched pair test of proportion.

Table 16a: Case control analysis of Change in Depression/Anxiety Score from Baseline to Post Intervention for those who had a score >=10 at the beginning of the intervention.

Category	Type	Statistics	Baseline	Post Intervention	Change	P-Value for difference
Anxiety	Cases	N	xxxxx	xxxxx	xxxxx	
		Mean (SD)	xx.x (xx.xx)	xx.x (xx.xx)	xx.x (xx.xx)	
		Median	xx.x	xx.x	xx.x	
		Min, Max	xx; xx	xx; xx	xx; xx	
	Control	N	xxxxx	xxxxx	xxxxx	
		Mean (SD)	xx.x (xx.xx)	xx.x (xx.xx)	xx.x (xx.xx)	0.xxxx
		Median	xx.x	xx.x	xx.x	
		Min, Max	xx; xx	xx; xx	xx; xx	
Depression	Cases	N	xxxxx	xxxxx	xxxxx	
		Mean (SD)	xx.x (xx.xx)	xx.x (xx.xx)	xx.x (xx.xx)	
		Median	xx.x	xx.x	xx.x	
		Min, Max	xx; xx	xx; xx	xx; xx	
	Control	N	xxxxx	xxxxx	xxxxx	
		Mean (SD)	xx.x (xx.xx)	xx.x (xx.xx)	xx.x (xx.xx)	0.xxxx
		Median	xx.x	xx.x	xx.x	
		Min, Max	xx; xx	xx; xx	xx; xx	

**   Case: The subjects who are screen positive by ASHA.*

***   Control: The subjects who are screened negative by ASHA but positive by study interviewer.*

P-Value is calculated based chi-square independent sample test of proportion.

Table 16b: Case control analysis for health service utilization

Proportion of subject used health services

Category	Baseline	Post Intervention	P-Value
Case*	xxxx	xxxx	0.xxx
Control**	xxxx	xxxx	

Health Service used: At baseline: Response from the question "Did you get treated for that mental disorder?"
At Post Intervention: Response from the question "Did you get treated for your mental health condition as indicated by ASHA/Interviewer?"

* *Case: The subjects who are screen positive by ASHA.*

** *Control: The subjects who are screened negative by ASHA but positive by study interviewer.*

P-Value is calculated based chi-square independent sample test of proportion.

Table 17: Subject Referrals during ASHA screening

Screened by		(N*=xxxxxx) n(%)
ASHA	Out of those Screened by ASHA Proportion of subject referred to the PHC doctor	xxxx (xx.xx)
	Out of those referred to PHC doctors Proportion of subjects actually visited doctors	xxxx (xx.xx)
	Out of those visited doctors Proportion of subjects appropriately prescribed anti-depressant or anxiolytic	xxxx (xx.xx)
	Out of those visited doctors Proportion of subjects appropriately referred by the PHC doctor for more	xxxx (xx.xx)

**N= overall population screened by ASHAs.*
