# Supplementary material for: The Systematic Medical Appraisal Referral and Treatment Mental Health Project: Quasi-Experimental Study to Evaluate a Technology-Enabled Mental Health Services Delivery Model Implemented in Rural India
Source: J Med Internet Res. 2020 Feb 27;22(2):e15553. doi: 10.2196/15553 (PMC7068463; doi:10.2196/15553)
Supplement: Multimedia Appendix 2 [file jmir_v22i2e15553_app2.pdf]

## Supplementary analyses

Post-intervention data collection: Follow-up data were collected from those individuals who were identified as screen positive during the baseline survey, but were assessed as screen negative by the ASHAs. This group did not receive the full technology-enabled mental health services delivery intervention, although they were exposed to the anti-stigma campaign and had received initial screening by ASHAs. (**Supplementary Figure 1**). Sociodemographic characteristics of that group are given in **Supplementary Table 1**.

The baseline survey conducted by the trained interviewers included 22,377 adults (80.3% of the total estimated eligible population) and they identified 1171 screen positive individuals (5.2%). Out of 1171, 150 were also screened positive by ASHAs. Out of the remaining 1021, who were screened positive by interviewers, but were not screened positive by ASHAs, 920 were screened at the end of the study during the post-intervention data collection. Only 17 out of those 920 (1.9%, 95%CI 1.0-2.7) individuals reported using any mental health services for their symptoms even after being advised to do so by the interviewers. Differences in anxiety and depression scores for those screened positive by interviewers at baseline and then reinterviewed at the end of the study are given in **Supplementary Table 2**.

**Supplementary Tables 3a-e** show the predictive models for mental health services use adjusted for intervention status and different socio-demographic covariates.

**Supplementary Figure 1: Diagram showing population contacted and interviewed at each stage amongst those who were screened positive by interviewers**

Population on whom pre-stigma assessment was done by interviewers [N=1576, across two village

Anti-stigma campaign rolled out in the whole community

Population on whom detailed **baseline** data was collected by interviewers [N=22377]

Population screened positive by interviewers [N=1171]

No intervention delivered

Population who were screened negative or were not traceable during ASHA screening [N=1021]

No intervention delivered

Population on whom data was collected at post-intervention [N= 920/1021 = 90.1%]

**Supplementary Table 1: Socio-demographic and health characteristics of study population who were screened positive by interviewers only**

| **Characteristic** | **Baseline Interviewer screened positive patients (N=1171)** |
| --- | --- |
| Age(Years) | |
| Mean (SD) | 50.1 (16.81) |
| Range | 18; 98 |
| | |
| Gender | |
| Female | 763 (65.2%) |
| Male | 408 (34.8%) |
| | |
| Occupation | |
| Unorganized Sector [a] | 549 (46.9%) |
| Organized Sector [b] | 241 (20.6%) |
| House wife/Retired | 21 (1.8%) |
| Other [c] | 360 (30.7%) |
| | |
| Education | |
| Graduate/Post Graduate | 20 (1.7%) |
| High School | 104 (8.9%) |
| No School | 547 (46.7%) |
| Others [d] | 8 (0.7%) |
| Primary School | 492 (42.0%) |
| | |
| Marital Status | |
| Currently Married | 864 (73.8%) |
| Never Married | 49 (4.2%) |
| Separated/ Divorced/ Widowed | 258 (22.0%) |
| | |

a- Agricultural labourer, Manual labourer, skilled worker, farmer and business are reported under unorganized sector
b- All regular salaried jobs were part of organized sector
c- Includes students, those searching for jobs, those unable to work because of illness and old age
d- Those pursuing vocational training

**Supplementary Table 2: Scores on anxiety (Generalized Anxiety Disorder – 7 item) and depression scales (Patient Health Questionnaire- 9 item) for those screened positive by interviewers and then reinterviewed at post-intervention**

| | **Anxiety** | | | |
|---|---|---|---|---|
| | **Normal** | **Mild** | **Moderate** | **Severe** |
| **category** | **0-4** | **5-9** | **10-14** | **>=15** |
| Intervention Positive Baseline (N=1171) | 474 (40.5%) | 384 (32.8%) | 228 (19.5%) | 85 (7.3%) |
| Intervention Positive Baseline (N=1060) | 905 (85.4%) | 119 (11.2%) | 20 (1.9%) | 16 (1.5%) |
| | **Depression** | | | |
| | **Normal** | **Mild** | **Moderate** | **Severe** |
| **category** | **0-4** | **5-9** | **10-14** | **>=15** |
| Intervention Positive Baseline (N=1171) | 242 (20.7%) | 383 (32.7%) | 372 (31.8%) | 174 (14.9%) |
| Intervention Positive Baseline (N=1060) | 843 (79.5%) | 135 (12.7%) | 54 (5.1%) | 28 (2.6%) |

**Supplementary Table 3a: Mental health services use adjusted for intervention and age**

| label | Odds ratio | 95% Lower Limit | 95% Upper Limit | P-value |
|---|---|---|---|---|
| Intervention | | | | |
| *Pre-intervention* | 1.0 | | | |
| *Post-Intervention* | 134.74 | 89.86 | 202.04 | <.0001 |
| Age | | | | |
| *<=29 years* | 1.0 | | | |
| *>=30 and <=59 years* | 1.09 | 0.65 | 1.81 | 0.75 |
| *>=60* | 1.33 | 0.78 | 2.27 | 0.29 |

**Supplementary Table 3b: Mental health services use adjusted for intervention and education**

| label | Odds ratio | 95% Lower Limit | 95% Upper Limit | P-value |
|---|---|---|---|---|
| Intervention | | | | |
| *Pre-intervention* | 1.0 | | | |
| *Post-Intervention* | 133.38 | 89.03 | 199.82 | <.0001 |
| Education | | | | |
| *No formal education* | 1.0 | | | |
| *Some formal education* | 1.06 | 0.78 | 1.45 | 0.71 |

**Supplementary Table 3c: Mental health services use adjusted for intervention and gender**

| label | Odds ratio | 95% Lower Limit | 95% Upper Limit | P-value |
|---|---|---|---|---|
| Intervention | | | | |
| *Pre-intervention* | 1.0 | | | |
| *Post-Intervention* | 133.64 | 89.19 | 200.24 | <.0001 |
| Gender | | | | |
| *Male* | 1.0 | | | |
| *Female* | 1.13 | 0.81 | 1.57 | 0.49 |

**Supplementary Table 3d: Mental health services use adjusted for intervention and marital status**

| label | Odds ratio | 95% Lower Limit | 95% Upper Limit | P-value |
|---|---|---|---|---|
| Intervention | | | | |
| *Pre-intervention* | 1.0 | | | |
| *Post-Intervention* | 137.76 | 91.38 | 207.67 | <.0001 |
| Marital status | | | | |
| *Never married* | 1.0 | | | |
| *Currently married* | 0.31 | 0.09 | 1.02 | 0.05 |
| *Divorced/separated/widowed* | 0.25 | 0.07 | 0.86 | 0.03 |

**Supplementary Table 3e: Mental health services use adjusted for intervention and occupation**

| label | Odds ratio | 95% Lower Limit | 95% Upper Limit | P-value |
|---|---|---|---|---|
| Intervention | | | | |
| *Pre-intervention* | 1.0 | | | |
| *Post-Intervention* | 133.61 | 89.18 | 200.19 | <.0001 |
| Occupation | | | | |
| *Currently not working* | 1.0 | | | |
| *Currently working* | 0.89 | 0.64 | 1.26 | 0.52 |
